# Assessment of cardiac pathology by point-of-care ultrasonography performed by a novice examiner is comparable to the gold standard

**DOI:** 10.1186/1757-7241-21-87

**Published:** 2013-12-13

**Authors:** Christian Alcaraz Frederiksen, Peter Juhl-Olsen, Niels Holmark Andersen, Erik Sloth

**Affiliations:** 1 Department of Cardiology, Aarhus University Hospital, Skejby, Denmark; 2 Department of Anesthesiology and Intensive Care, Aarhus University Hospital, Skejby, Denmark; 3 Department of Clinical Medicine, Faculty of Health, Aarhus University, Aarhus, Denmark

**Keywords:** Point-of-care, Ultrasonography, Echocardiography, Bedside, Heart disease

## Abstract

**Background:**

The aim of the study was to compare the diagnostic accuracy of point-of-care cardiac ultrasonography performed by a novice examiner against results from a specialist in cardiology with expert skills in echocardiography, with regard to the assessment of six clinically relevant cardiac conditions in a population of ward patients from the Department of Cardiology or the Department of Cardiothoracic Surgery.

**Methods:**

Cardiac ultrasonography was performed by a novice examiner at the bedside and images were interpreted in a point-of-care context with dichotomous outcomes (yes/no). Six outcome categories were defined: 1) pericardial effusion (≥10 mm), 2) left ventricular dilatation (≥62 mm), 3) right ventricular dilatation (≥42 mm or ≥ left ventricular diameter), 4) left ventricular hypertrophy (≥13 mm), 5) left ventricular failure (EF ≤ 40%), 6) aortic stenosis (maximum flow velocity ≥3 m/s). The examiner was blinded to the patients’ medical history and results from previous echocardiographic examinations. Results from the interpreted point-of-care ultrasonography examination were compared with echocardiographic diagnosis made by a specialist in cardiology.

**Results:**

A total of 102 medical and surgical patients were included. Assessments were made in six categories totalling 612 assessments. There was agreement between the novice examiner and the specialist in 95.6% of the cases; overall sensitivity was 0.91 and specificity was 0.97. Positive predictive value was 0.92 and negative predictive value was 0.97. Kappa statistics showed good agreement between observers (κ=0.88).

**Conclusions:**

This study showed that a novice examiner was able to detect common and significant heart pathology in six different categories with good accuracy using POC ultrasonography.

## Background

Severe heart disease is a serious entity worsening outcome in the emergency department [1,2], during surgery and anaesthesia [3,4], and in the critical care setting [5,6].

Traditionally, physicians have relied on medical history, physical examination, electrocardiography and chest radiographs to screen for cardiovascular pathology. However, the diagnostic performance of these techniques has been questioned [7-9].

Full evaluation by an expert in cardiology including a diagnostic transthoracic echocardiography (TTE) offers a comprehensive assessment of the most severe heart diseases [10]. However, due to equipment costs and extensive time and training resources required to complete a full TTE, this examination is seldom readily available at the bedside upon admission, hence delaying information about cardiovascular function.

To address problems associated with limited availability of a full TTE, several point-of-care (POC) ultrasonography protocols have emerged [11-13]. POC ultrasonography can be characterized as a real time examination brought to the bedside of the patient and performed by the provider, usually a non-cardiology trained clinician, and with limited training in ultrasonography. In contrast to a full standard TTE it is important that the examination is completed within a limited amount of time and that outcomes are well defined clinical problems with a dichotomous (yes/no) response [14].

The development of POC protocols was facilitated by advances in technology, providing high quality equipment with great portability [15], and for cardiopulmonary optimization, the Focus Assessed Transthoracic Echocardiography (FATE) protocol [16] was developed in the early 1990′s. Today, the FATE protocol is part of the standard curriculum in a limited number of European teaching hospitals and has been shown to be applicable by experts in an ICU setting and during cardiac surgery [16,17]. It has also been applied to patients in a seated position using a pocket device with high image quality and low time consumption [15]. Most recently POC cardiac ultrasonography has been shown to be applicable among novice examiners with no previous experience in ultrasonography, producing high numbers of images suitable for interpretation in healthy subjects [18].

If POC cardiac ultrasonography is to be used among novice examiners and non-cardiologists, knowledge about sensitivity and specificity is required. Furthermore, the novice user is required to be able to recognise normal images, be able to provide reproducible ultrasonography standard views, be able to recognize pathology and to relate the findings to the clinical context. Another key element of POC ultrasonography is to be able to detect cardiac pathology with a high degree of reliability.

The purposes of this study was to compare the diagnostic accuracy of point-of-care cardiac ultrasonography performed by a novice examiner against results from a specialist in cardiology with expert skills in echocardiography with regard to the assessment of six clinically relevant cardiac conditions in a population of ward patients from the Department of Cardiology or the Department of Cardiothoracic Surgery.

## Methods

### Study population & selection process

The study was performed in accordance with the Helsinki Declaration and informed consent was obtained from all patients. The study was reviewed by the The Central Denmark Region Committees on Health Research Ethics and due to the design of the study it was exempt from further ethical approval.

**Figure 1:**
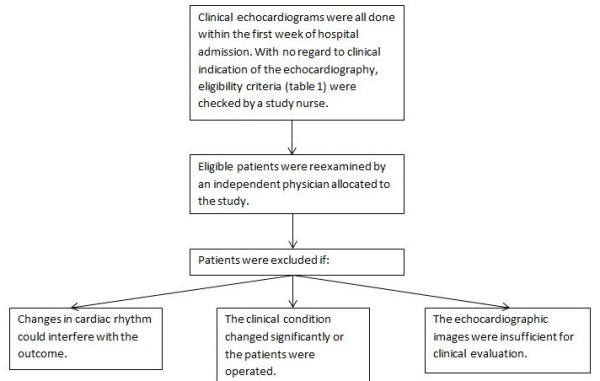
Schematic overview of the selection process of included patients.

Patients undergoing a standard echocardiographic examination at the Department of Cardiology were eligible for inclusion. The selection process was performed by an independent nurse and physician affiliated with the study. During data collection, patients were always screened and included consecutively in order to avoid selection bias. All eligible patients were assessed on all study days. In addition to Table 1, the selection process is outlined in Figure 1. The clinical presentation of enrolled patients was characterized by hemodynamic stability and no severe distress symptoms. All patients were admitted at the Department of Cardiology or the Department of Thoracic Surgery. After the patient consented to participate; the novice examiner performed a POC ultrasonography examination at the bedside completely blinded to the selection process.

**Table 1:** Criteria used in the screening of patients for eligibility

**Patients eligible for inclusion fulfilled one of the following criteria**	
Pericardial exudate	≥ 10 mm
Left Ventricle (end-diastolic diameter)	≥ 62 mm
Right Ventricle (end-diastolic diameter)	≥ 42 mm or ≥ LVEDD
Myocardial thickness	≥ 13 mm
Ejection fraction	≤ 0.40
Aortic stenosis (maximum flow velocity)	≥ 3 m/s
Normal standard echocardiography	

### Novice examiner

Prior to study initiation the novice examiner had the following experience:

• One year of training in internal medicine and two years of training in anaesthesiology, this includes emergency medicine and intensive care medicine in Denmark.

• A one day predominantly hands on workshop covering basic imaging techniques and FATE views as well as teaching using a scenario based approach.

• In addition to the basic FATE course, a short introduction to continuous wave Doppler pressure estimation and the 5-chamber view was given.

• Approximately 50 POC cardiac ultrasonography examinations including 10 with supervision from an expert (level 3).

These prerequisites are similar to level 1 competence agreed by experts published in a variety of statement papers and is the minimum requirements for performing unsupervised POC cardiac ultrasonography [19,20].

### Equipment and data acquisition

A Vivid S6 (GE Healthcare, Horten, Norway) ultrasound system equipped with a M4S phased array transducer (1.5 – 4.5 MHz) with second harmonic imaging was used for data acquisition.

All patients underwent POC cardiac ultrasonography at the bedside. No guidance or supervision was provided for the novice examiner during image acquisition or interpretation throughout the study period.

POC ultrasonography included the following views: Subcostal 4-chamber, apical 4-chamber, apical 5-chamber including continuous wave Doppler of the left ventricular outflow tract, parasternal long- and short-axis. Raw data were digitally stored in cineloop format defined by the R-wave in the corresponding electrocardiogram for off-line analyses. The examination was initially performed with patients placed in the supine position, and if the condition of the patient allowed, image acquisition was also performed in the left lateral position.

### Data analyses

**Figure 2:**
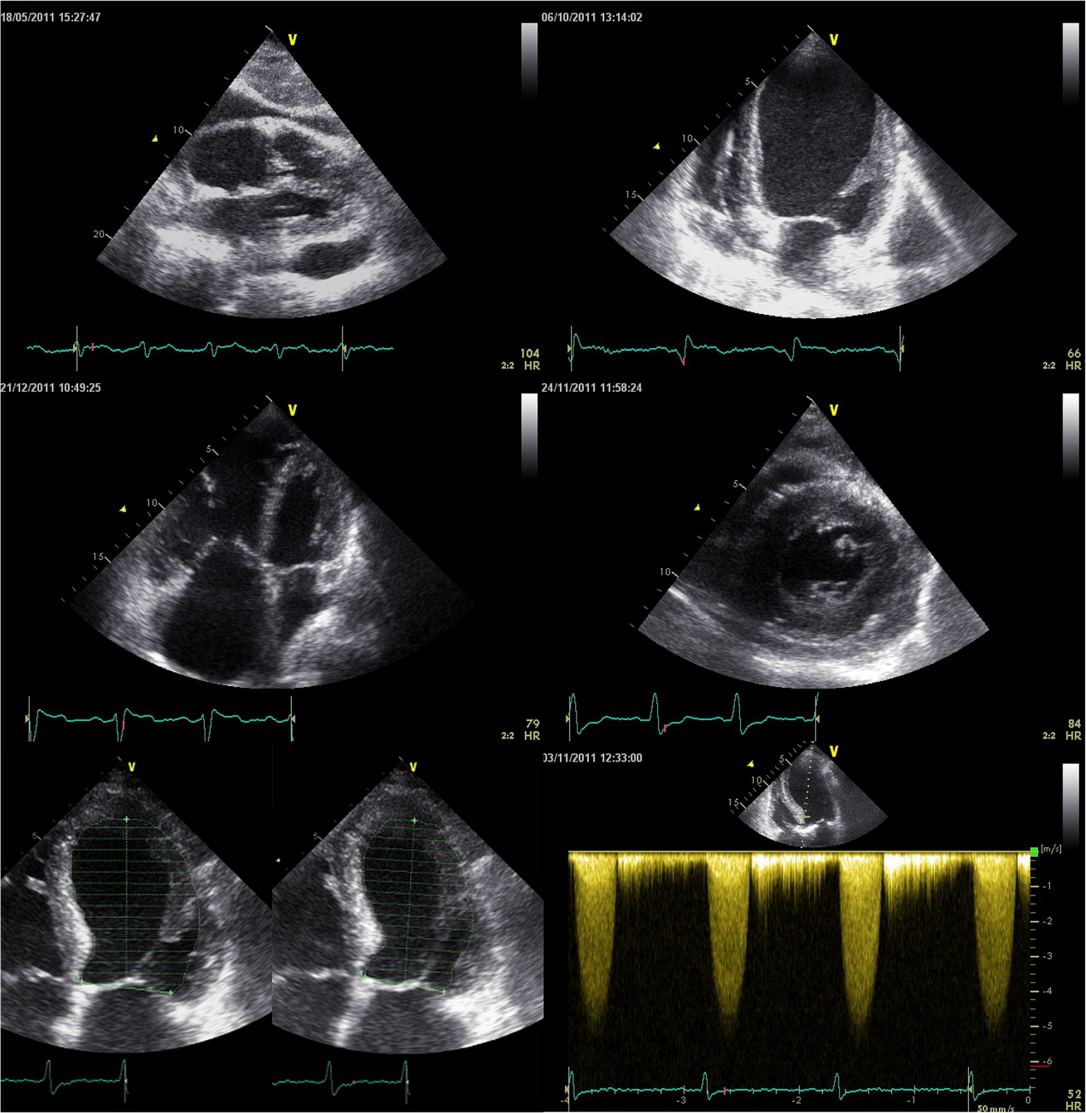
**Examples of severe pathology detected by POC ultrasonography at the bedside.** Images were obtained by the novice examiner. *Upper left panel:* Pericaldial exudates. *Upper right panel:* Left ventricular dilation. *Middle left panel:* Right ventricular dilation. *Middle right:* Myocardial hypertrophy. *Lower left:* Impaired ejection fraction. *Lower right:* Aortic stenosis.

All examinations were interpreted by the novice examiner who had performed the image acquisition, and the novice examiner was blinded to results from previous echocardiography examinations. The interpretations and estimates were done as post-examination analyses using EchoPac software (GE Healthcare, Horten, Norway). Images were interpreted and categorised according to dichotomous outcomes in the following six categories (Table 1 & Figure 2):

• Pericardial effusion (≥10 mm). The severity was measured as simple two-dimensional calliper assessments of the largest echo-free zone between the pericardial layers in any cardiac view.

• Left ventricular dilatation (≥62 mm). The left ventricle diameter was measured at the end of diastole in the parasternal long axis view or the apical 4-chamber view, at the tip of the mitral leaflets, at the interface between blood and inner ventricular wall.

• Right ventricular dilatation (≥42 mm or ≥ left ventricular diameter). The right ventricle diameter was measured at the end of diastole in the apical 4-chamber view, at the tip of the tricuspid leaflets, at the interface between blood and inner ventricular wall.

• Left ventricular hypertrophy (≥13 mm). The left ventricular wall thickness was measured at the end of diastole in the parasternal long axis view or the parasternal short axis view, at the base of the ventricle on the interventricular septum.

• Left ventricular failure (EF ≤ 40%). Left ventricular ejection fraction was measured by estimating the left ventricular volume during systole and diastole using the method of discs in the apical 4-chamber view. In the case of suboptimal image quality eyeballing was used.

• Aortic stenosis (maximum flow velocity ≥3 m/s). In the case of aortic stenosis, the severity was measured using a combination of two-dimensional observations from the parasternal long axis view and the apical 5-chamber view and spectral Doppler analyses of the blood flow through the aortic valve.

The cut-off values of outcome variables are primarily selected based on current recommendations from the European and American societies of echocardiography [21,22]. Also institutional practices were taken into account. M-mode was not used in order to avoid angular problems and all dimensions were measured using the calliper function.

### Gold standard reference

In order to establish gold standard results for all examinations a second interpretation was made by a specialist in cardiology with expert skills in echocardiography. This expert examiner reviewed all the images obtained by the novice examiner and categorised all patients according to the dichotomous outcomes in the pre-defined criteria. In the case of sub-optimal image quality or doubt about the outcomes, the expert also had full access to images and interpretations obtained from the original clinical echocardiogram done by another cardiologist. The expert was blinded to results made by the novice examiner.

### Statistical analyses

Diagnostic performance was estimated with sensitivity and specificity. Predictive values were calculated and kappa statistics were applied. Comparison of results between the novice examiner and the specialist was estimated by McNemar’s test. Tests and calculations were performed using Stata 11.0 software (StataCorp LP, Texas, USA).

## Results

A total of 102 medical and surgical patients were included. The mean age of patients was 63.2 ±16.4 years and 31% were females. Information on discharge diagnosis is shown in Table 2.

**Table 2:** Discharge diagnosis of all included patients categorized according to the primary clinical problem(s)

**Diagnosis**	**(n = 102)**
Ischemic heart disease	35 (34.3%)
Aortic stenosis	20 (19.6%)
Endocarditis	13 (12.7%)
Atrial fibrillation	6 (5.9%)
Venous thromboembolism	5 (4.9%)
Cardiomyopathy	5 (4.9%)
Mitral regurgitation	5 (4.9%)
Arrhythmia	4 (3.9%)
Myopericarditis	4 (3.9%)
Pulmonary hypertension	3 (2.9%)
Hypertrophic cardiomyopathy	3 (2.9%)
Aortic regurgitation	2 (2.0%)
Aortic dissection	2 (2.0%)
Atrial septal defect	1 (1.0%)
Arrhythmogenic right ventricular dysplasia	1 (1.0%)
Amyloidosis	1 (1.0%)

Atrial fibrillation is separated from other arrhythmias.

Overall, 102 assessments were made in six categories totalling 612 assessments. Pericardial effusion was present in 15 patients, left ventricular dilatation was present in 22 patients, right ventricular dilatation was present in 19 patients, left ventricular hypertrophy was present in 27 patients, left ventricular failure was present in 38 patients and aortic stenosis was present in 33 patients. A detailed description of pathological findings is shown in Table 3.

**Table 3:** Detailed description of dichotomous results is shown

**Expert findings**	**(n = 102)**
No pathology	19 (18.6%)
Left ventricular failure + left ventricular dilatation	9 (8.8%)
Aortic stenosis	8 (7.8%)
Left ventricular hypertrophy + aortic stenosis	8 (7.8%)
Left ventricular failure	7 (6.9%)
Right ventricular dilatation	7 (6.9%)
Pericardial effusion	5 (4.9%)
Left ventricular hypertrophy	5 (4.9%)
Left ventricular dilatation + right ventricular dilatation + left ventricular failure	5 (4.9%)
Left ventricular hypertrophy + left ventricular failure	5 (4.9%)
Pericardial effusion + left ventricular hypertrophy + aortic stenosis	4 (3.9%)
Left ventricular dilatation + left ventricular failure + aortic stenosis	4 (3.9%)
Pericardial effusion + aortic stenosis	3 (2.9%)
Right ventricular dilatation + left ventricular failure	3 (2.9%)
Left ventricular dilatation	2 (2.0%)
Right ventricular dilatation + left ventricular hypertrophy + aortic stenosis	1 (1.0%)
Pericardial effusion + left ventricular hypertrophy + left ventricular failure	1 (1.0%)
Pericardial effusion + left ventricular failure + aortic stenosis	1 (1.0%)
Left ventricular dilatation + right ventricular dilatation + left ventricular failure + aortic stenosis	1 (1.0%)
Pericardial effusion + right ventricular dilatation + left ventricular hypertrophy + aortic stenosis	1 (1.0%)
Left ventricular hypertrophy + left ventricular failure + aortic stenosis	1 (1.0%)
Right ventricular dilatation + aortic stenosis	1 (1.0%)
Left ventricular dilatation + left ventricular hypertrophy + left ventricular failure	1 (1.0%)

Assessments were made from images obtained by a novice examiner by an expert in cardiology and echocardiography.

There was agreement between the novice examiner and the specialist in 95.6% of the cases. The overall sensitivity was 0.91 and specificity was 0.97. Positive predictive value was 0.92 and negative predictive value was 0.97. Kappa statistics showed good agreement between observers (κ=0.88). McNemar’s test showed no difference between the novice examiner and the specialist (P = 1.00). Detailed results from each category are shown in Table 4.

**Table 4:** Diagnostic performance parameters comparing FATE bedside examination performed by a novice examiner with results of an expert in cardiology and echocardiography

	**PE**	**LVEDD**	**RVEDD**	**MT**	**EF**	**AS**
**True positive**	15	20	16	23	35	31
**True negative**	86	79	79	74	59	68
**False positive**	1	1	4	1	5	1
**False negative**	0	2	3	4	3	2
**Sensitivity**	1.00	0.91	0.84	0.85	0.92	0.94
**- 95% CI**	(0.78-1.00)	(0.71-0.99)	(0.60-0.97)	(0.66-0.96)	(0.79-0.98)	(0.80-0.99)
**Specificity**	0.99	0.99	0.95	0.99	0.92	0.99
**- 95% CI**	(0.94-1.00)	(0.93-1.00)	(0.88-.099)	(0.93-1.00)	(0.83-0.97)	(0.92-1.00)
**PPV**	0.94	0.95	0.80	0.96	0.88	0.97
**- 95% CI**	(0.70-1.00)	(0.76-1.00)	(0.56-.094)	(0.79-1.00)	(0.73-0.96)	(0.84-1.00)
**NPV**	1.00	0.98	0.96	0.95	0.95	0.97
**- 95% CI**	(0.96-1.00)	(0.91-1.00)	(0.90-.099)	(0.87-0.99)	(0.87-0.99)	(0.90-1.00)
**Kappa**	0.96	0.91	0.78	0.87	0.83	0.93

PE (Pericardial exudates), LVEDD (Left Ventricle end-diastolic diameter), RVEDD (Right Ventricle end-diastolic diameter), MT (Myocardial thickness), EF (Ejection fraction), AS (Aortic stenosis), PPV (Positive predictive value), NPV (Negative predictive value), 95% CI (95% confidence interval).

## Discussion

In this study the results showed very good agreement between a novice examiner performing POC cardiac ultrasonography and reference values when dealing exclusively with dichotomous outcomes.

The reason for choosing dichotomous outcomes instead of continuous variables relates to the fundamental ideas of POC ultrasonography and also the clinical reality in which POC ultrasonography is used. In the context of anaesthesiology, critical care or emergency medicine it is sufficient to identify the presence of normal or reduced EF. In these settings it is not clinically relevant to sub-categorize EF in intervals of e.g. 5-10%, since this discrimination will have no immediate implications for the clinical management of the patient. Similarly, it is often not clinically relevant to discriminate between small differences in left ventricular end-diastolic diameters, but it is important to know if the left ventricle is severely dilated or not [23,24].

Results in most pathology categories showed very good agreement, although some categories performed better than others. Sensitivity with regard to RV diameter and myocardial thickness was 0.84 and 0.85, respectively, whereas sensitivities in the remaining categories were all above 0.9. The reason for suboptimal diagnostic performance when assessing the RV can be explained by the complex geometry of the ventricle and incomplete visualization by 2-dimensional ultrasonography. Suboptimal diagnostic performance when measuring myocardial thickness can be attributed to the fact that many of the cases were borderline hypertrophic. This has a major impact on the results due to the dichotomous categorization. The same phenomenon of borderline measurements on the wrong side of the cut-off accounts for almost every false positive and false negative result. Each of the false results was reviewed and almost every one proved to be borderline cases. Unfortunately, we do not have data on the continuous variables assessed by the expert, and hence it is not possible to provide further details on these borderline cases. However, the reason for most of the borderline cases can be attributed to the fact that significant pathology in one category (e.g. left ventricular failure) and insignificant pathology (e.g. pericardial effusion of 7 mm) in another category were allowed into the study, introducing difficult estimations close to the cut-off values. In contrast, patients with only mild pathology in only one category were not allowed into the study reducing the number of borderline cases.

An aortic stenosis often represents a clinical challenge in the context of anaesthesiology, critical care or emergency medicine. In this study we found a very high sensitivity and specificity probably because of a relatively low number of borderline cases. However, the detection and quantification of an aortic stenosis can be challenging especially with concomitant left ventricular failure. Thus, to further increase the diagnostic accuracy when assessing aortic valves it should be recommended to supplement the Doppler recordings with simple 2D imaging of the valve.

### Clinical implications

POC ultrasonography is already common in many clinical settings and it is predicted that the modality can decrease medical errors and provide efficient real-time diagnoses [25].

In the cardiopulmonary area there is increasing evidence that novice examiners can learn focused POC ultrasonography protocols and implement them in a variety of medical contexts [25]. An obvious setting for POC cardiopulmonary ultrasonography includes emergency medicine, critical care, anaesthesiology, and medical wards. Patients diagnosed and treated in these settings will very often have multiple pathologies in the heart. This was also the case for many of the patients in the current study which strengthens its validity.

The equipment used in the current study was a high-end cart-based system with the purpose of optimal conditions for the novice examiner. However, bedside POC ultrasonography in the future will probably be performed on pocket sized systems and studies investigating novice examiners using these relatively new machines have already been published showing diagnostic accuracy superior to a normal clinical examination [26,27].

Considering the present and also other recent studies, it seems reasonable to allow novice examiners to perform and interpret POC ultrasonography. However, it should always be remembered that the ultrasonographic findings can never stand alone. Whether or not an ultrasonographic finding represents hemodynamically significant pathology or not requires a full clinical evaluation and possibly further testing or imaging.

### Study design

During the design phase of the current study the number of novice examiners performing POC cardiac ultrasonography was thoroughly considered. The statistically and methodological optimal setting would probably have been to include 10–20 novice examiners from the same department and have them evaluate all the patients. This would contribute information on variability in skill among examiners. However, several patients included were suffering from severe heart disease and some of them even dying, so it was not ethically feasible to subject the patients to ultrasonography by 10-20 different novice examiners.

The fact that one novice examiner evaluated all the patients may raise the question as to whether this person was truly a novice at the end of the study. However, experts agree that level 1 or novice skills in POC cardiac ultrasonography requires between 150–300 studies [19,20]. This infers that the novice examiners from the current study could still be considered novice at the end of the study, and it seems reasonable that other novices would be able to achieve the same results. However, since the results from the current study lack some degree of external validity, the findings can be seen as a proof of concept and further studies are needed. Since interpretational skills primarily relies on pattern recognition and is somewhat independent from technical skills, it seems reasonable that a future study could overcome the ethical concerns in the current study by allowing 10–20 novice examiners to review echocardiographic images obtained in the current study for the purpose of more valid assessment of sensitivity and specificity.

### Limitations

The novice examiner performing all the examinations in the current study was motivated to acquire skills in POC ultrasonography and therefore questions about selection bias can be raised. However, the novice examiner did have any special skills or prerequisites not obtainable by other physicians and, in general, young physicians are usually highly inclined to POC ultrasonography.

Pleural scanning is usually part of the FATE protocol. However, we did not include assessment of the pleural cavity is this study, as no reference assessments were available from the reference echocardiographic images. In addition, simple colour Doppler assessment of valvular regurgitations was not part of the POC protocol used in the present study, since most examiners are non-cardiologist with limited experience. However, as skills increase this could be a relevant addition in future studies since this modality is now available even in pocket sized devices.

The medical and surgical patients included were screened according to the criteria listed in Table 1, and were thus a selected group of patients with a large proportion of ultrasonographic pathology. However, patients with completely normal findings during standard echocardiography were also included in order to reduce confounding. In addition, hemodynamically stable ward patients were studied, which entails that the results cannot readily be extrapolated to unstable patients in the emergency department or in the intensive care unit.

The pre-defined outcome categories used in this study were chosen because they represent clinically important situations. However, assessment of these six categories does not represent a total hemodynamic evaluation. This would require a full specialist echocardiography and sometimes even invasive measures.

Nevertheless, the cut-off values chosen for this study were needed to operate with dichotomous outcomes which seem relevant in the context of anaesthesiology, critical care or emergency medicine where POC ultrasonography is predominant.

It is a well-known fact that even when experts perform full standard TTE considerable intra- and inter-observer variation has to be taken into account. In addition, the expert responsible for gold standard assessment in this study did not perform the examinations himself. These two facts may have contributed to additional variation in the results. In addition, full standard TTE is sometimes hampered by poor image quality requiring transesophageal examination. Only two patients were excluded due to poor image quality in this study plausibly representative of everyday clinical practice.

## Conclusions

This study showed that a novice examiner was able to detect common and significant heart pathology in six different categories with good accuracy using POC ultrasonography. Interpretation of the results has limitations and further studies must be conducted in order to assess whether limited systematic education leads to sufficient skills and whether implementation of the modality leads to an improved patient outcome.

## Abbreviations

TTE: Transthoracic echocardiography; POC: Point-of-care; FATE: Focus assessed transthoracic echocardiography.

## Competing interests

The authors declare that they have no competing interest.

## Authors’ contributions

CF, PJ, NA, and ES participated in the design of the study. CF and PJ carried out the data acquisition. CF and NA performed the data analysis and CF performed the statistical analysis. CF and ES drafted the manuscript. All authors read, revised and approved the final manuscript.
